# Optical Indicators based on Structural Colored Polymers

**DOI:** 10.1002/advs.202200399

**Published:** 2022-03-11

**Authors:** Yari Foelen, Albert P. H. J. Schenning

**Affiliations:** ^1^ Department of Chemical Engineering and Chemistry Eindhoven University of Technology Den Dolech 2 Eindhoven 5600 MB The Netherlands; ^2^ Institute for Complex Molecular Systems Eindhoven University of Technology Den Dolech 2 Eindhoven 5600 MB The Netherlands; ^3^ SCNU‐TUE Joint Laboratory of Device Integrated Responsive Materials (DIRM) South China Normal University Guangzhou Higher Education Mega Center Guangzhou 510006 China

**Keywords:** optical indicators, photonic materials, structural color, time temperature integrators

## Abstract

Polymer indicators are autonomous responsive materials that provide an optical signal of a specific exposure in time. This review describes the different polymer systems utilized to obtain indicators based on structural color. Structural color originates from the interaction of light with a periodic nanostructured polymer which causes a specific wavelength to be reflected. This reflected light can be used for fabricating battery‐free indicators that show visible structural color changes upon exposure to a stimulus or analyte. In this review, the typical structural color response types categorized by stimulus are discussed and compared. Furthermore, the steps toward possible applications of optical indicators based on structural colored polymers are outlined.

## Graphical abstract

Optical indicators are autonomous responsive materials that provide an optical indication of a specific exposure history. This review describes the different polymer systems utilized to obtain a photonic response based on structural color. The response types are discussed and compared, which allows for an extensive comparison of methods applied in the broad field of responsive photonic polymers.

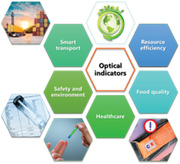

## 1 Introduction

Structural colored polymers have many applications as autonomous responsive photonic systems of which optical indicators are an often‐overlooked subset that are able to offer applications as information carriers and tracking systems.^[^ ^1^ ^]^ Optical indicators address the increasing societal need to monitor and track exposure conditions for healthcare, nutrition, safety, and transport.^[^ ^2^ ^]^ In healthcare, the need for cheap, simple indicator systems that can be used at home is indisputable and is still an evolving research area. For consumable products such as food, sensing and tracking are imperative operations to manage and monitor conditions during storage and transport. This provides a better guarantee for food quality and safety.^[^ ^3^ ^]^ For nonperishable, fragile products, pressure indicators give an insight in the handling history throughout transport. Personal safety to prevent exposure to, for instance, UV‐radiation requires efficient indicator systems. Therefore, an increasing demand for the development of new optical materials and methods must be met by research innovations. These novel materials are required to be tunable in response selectivity and sensitivity to provide indicators specifically tailored to the application and with an ease of use for in field deployment. Additionally, these indicators have to be available at low cost for rapid diagnostics and quality control applications.

One appealing approach for creating these indicators is through structural colored polymers. This category of responsive photonic materials allows for a facile optical readout of the signal by eye or by a (smartphone) camera. In general, the response of these materials is versatile and programmable at a molecular level to deliver a macroscopic optical response. Furthermore, the production procedures are generally cheap manufacturing methods, such as printing or coating, as many of these nanostructured systems form by self‐assembly. These polymer materials are highly durable, stable under most conditions, and they respond autonomous, making them battery‐free.

**Figure 1:**
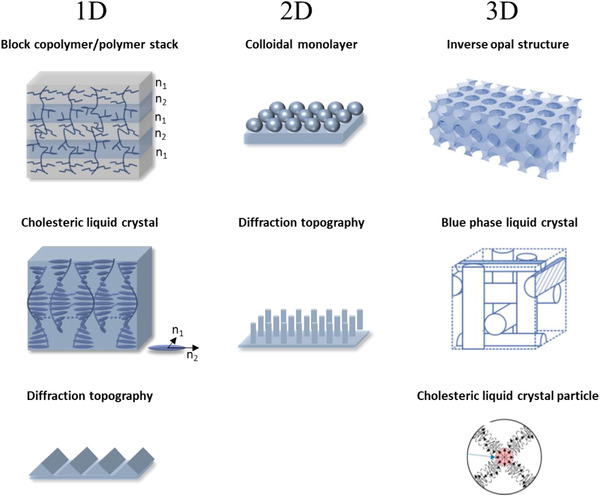
Some typical 1D, 2D, 3D structural color polymer systems that demonstrate a periodic alternation of the refractive index (*n*_1_, *n*_2_) at the nanoscale. 1D: block copolymers or multistacked polymer layers with indication of the refractive index changing by layers. Cholesteric LCs with the anisotropic refractive index indicated over one LC rod‐shaped molecule. 1D diffraction topography. 2D colloid monolayer and a 2D diffraction topography such as homogenous pillars. 3D: Inverse opal structure. Adapted with permission.^[^ ^21^ ^]^ Copyright 2020, Frontiers Media S.A. Blue phase LC. Adapted with permission.^[^ ^22^ ^]^ Copyright 2019, American Chemical Society. Cholesteric LC particle with helix alignment perpendicular to the particle surface. Adapted with permission.^[^^20^^]^ Copyright 2019, American Chemical Society.

Structural color originates from the interaction of light with a periodic alternation of the refractive index at the nanoscale which causes a specific wavelength to be reflected due to constructive interference.^[^ ^4^, ^5^ ^]^ The wavelength of the reflected light depends on the dimensions of the periodic nanostructure in the materials and the refractive indexes.^[^ ^6^ ^]^ A periodicity can emerge in either 1D, 2D, or 3D alternations of the refractive index which can be achieved by various polymer photonic systems (**Figure** **1**). 1D structural color can be created on a surface when nanostructures create a diffraction grating through the interference of the polymer and air interface.^[^ ^7^ ^]^ Thin film or multifilm 1D interference is created when light interferes with the refractive index alternations throughout the material. In polymers, multifilm interference is established through stacking of polymer layers^[^ ^8^ ^]^ or by self‐assembly of lamellar block copolymers^[^ ^9^ ^]^ (BCP). In cholesteric liquid crystal (LC) polymers, the interference of light is possible due to the anisotropic refractive index of LC rod‐shaped molecules.^[^ ^10^ ^]^ These LC molecules have a difference in the refractive index along the long axis of the molecule and in the orthogonal plane to this axis. Hence, a helical supramolecular structure causes a periodicity in the refractive index. 1D structural color systems demonstrate iridescence as the interference of light is angular dependent: the angle of the light pad also influences the observed layer spacing in 1D systems.^[^ ^11^, ^12^ ^]^ 2D structural color responsive polymers can be obtained from the diffraction of homogenous surface features or from a scattering effect when there is no order in the surface features.^[^ ^7^ ^]^ Similarly, a colloidal polymer monolayer on top of a polymer hydrogel is also consider a 2D effect.^[^ ^13^ ^]^ 3D structural color can be created by an inverse opal structure in polymer hydrogels.^[^ ^14^, ^15^ ^]^ Inverse opal hydrogels (IOH) are constructed by templating of monodisperse spheres. The self‐assembled colloidal crystals are embedded in a polymer hydrogel, whereafter the spheres are removed from the medium by pyrolysis or etching. 3D structural color can also be present in block copolymers that are used to self‐assemble multilayer particles that demonstrate a photonic reflectance.^[^ ^16^, ^17^ ^]^ The same holds for LC polymers, which can demonstrate 3D structural color in the complex blue phase^[^ ^18^, ^19^ ^]^ or in cholesteric LC polymer particles.^[^ ^20^ ^]^ 3D photonic polymers do not exhibit iridescence as the interference with light is not angular dependent.

**Figure 2:**
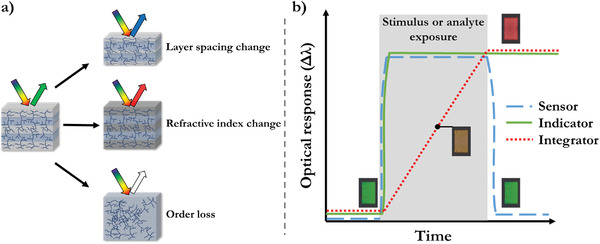
a) 1D polymer Bragg reflection of light by the different response mechanisms: lattice spacing, refractive index change, and order loss. b) Stimulus response type definitions based on the response behavior during and after exposure to an analyte or stimulus.

Responsive photonic polymers change their reflective properties upon a stimulus based on either change in the lattice spacing, order(s), and/or (surrounding) refractive index (**Figure** **2**a).^[^ ^23^, ^24^ ^]^ The optical response resulting from a change in layer spacing shows a red shift of the reflection band by expansion or a blue shift by contraction of the polymer. An increase of the refractive index difference will result in a red shift, and a decrease of this difference in a blue shift of the reflection band. A loss of order will result in a more transparent or scattering coating, either losing the constructive interference with light or reflecting all wavelengths.^[^ ^6^ ^]^

The stimulus or analytes that trigger a structural color response in polymer materials can be temperature, chemical exposure, pressure (or strain), and light. A general response is based on polymer expansion which causes a color difference by the change in lattice spacing. Many of the chemical indicators use a chemical analyte as a facilitator to induce solvent swelling or deswelling of the polymer. This swell–deswell dynamic allows for real time monitoring of a current state such as humidity with a hygroscopic polymer.^[^ ^25^ ^]^ Similarly, pH or ion monitoring in aqueous solution is, e.g., possible through a reversible volume change by the water uptake in response to the Donnan potential.^[^ ^26^, ^27^ ^]^

Most of the responsive photonic polymer systems are reversible and only allow for an instant optical readout. When the stimulus or analyte is taken away, the original structural color appears again, this is defined as a sensor. While some applications require a reversible response to provide an optical rendition of the present stimulus, it is often necessary to have an optical transcription of a certain exposure when the stimulus is no longer present. Throughout literature the terms “sensor” and “indicator” are often interchangeable, however, a strict definition is applied in this work. A sensor merely translates the signal of a stimulus into an optical response if the stimulus is present, while an indicator holds the response after the stimulus is no longer present. To obtain an indicator response, the polymers have to retain the changed structural configuration. The response is considered irreversible when at ambient conditions and by absence of the stimulus a certain past exposure can be deducted. Note that some polymer‐based indicators are reconfigurable and can be used multiple times after the execution of a specific recovery procedure.

A special case of indicator is an integrator (a portmanteau of integrating indicator), these indicators have multiple contributions to the response change such as a time component (Figure 2b). The term integrator is mostly used for time–temperature indicators as they integrate an isotropic temperature exposure over time. Though not commonly applied to other combinations of stimuli, the definition can still be applied to, for instance, analyte–time integrators that have slow exchange kinetics or a time‐dependent diffusion component in their response.^[^ ^28^ ^]^ This distinction is not consequently applied in literature and in most examples there is room for discussion as a response always has a time factor in the initial phase due to reaction kinetics. To adhere to the conventional term applied in literature, analyte‐responsive applications will be labeled as indicators in this review. An annotation about the response kinetics is provided when possible.

In general, the (temperature, concentration, pressure, light) range of response is limited for each type of indicator. Therefore, the optical response of indicators is always proportional to the saturation limits of the system. The sensitivity of detection is inversely correlated to the response range, primarily imposing a tradeoff between the detection limit and the detection range.

In this review, an overview of optical indicators and integrators based on structural colored polymers is presented with the focus on the response mechanism and the optical effect. Reversible‐responsive structural polymer systems are beyond the scope of this review, these sensors have been extensively described in other works.^[^ ^10^, ^24^, ^29^, ^30^ ^]^ For optical indicator and integrators not based on structural colors, the reader is also referred to other reviews.^[^ ^2^, ^31^, ^32^, ^33^ ^]^

By comparing different types of structural color optical indicators and integrators, a broad analysis lays out which common approaches are applied, where overlooked opportunities can unlock new research, and which limits still need to be overcome.

## 2 Temperature‐Responsive Optical Indicators

**Figure 3:**
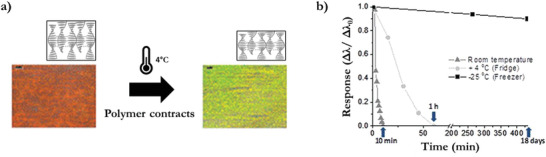
Solvent evaporation‐based TTI. a) Schematic representation of the TTI based on water evaporation: the water‐saturated polymer reflects a red color that shifts to green upon water release. b) Time‐dependent optical response to isothermal temperature exposure (room temperature, 4 and −25 °C). Reproduced with permission.^[^ ^25^ ^]^ Copyright 2012, American Chemical Society.

A temperature‐induced expansion or contraction of a photonic polymer causes a structural color change. This principle can be exploited in an irreversible manner by the temperature‐dependent evaporation of a solvent at a certain temperature. One example demonstrates a printable cholesteric LC film which is converted into a hygroscopic polymer salt. After water absorption, the time–temperature‐dependent desorption causes a contraction of the polymer through the irreversible loss of water above 0 °C (**Figure** **3**a). A time‐dependent blue shift happens in 10 min at room temperature or over an hour at 4 °C, resulting in a time–temperature integrator (TTI; Figure 3b).^[^ ^25^ ^]^

Photonic shape memory polymers employ the glass transition temperature (*T*_g_) as a polymer phase transition to enable a temperature response that accumulates over time. Above the *T*_g_, the polymer network can be deformed or compressed due to the low modulus. The deformation results in a change of the structural color and can be locked in by cooling below the *T*_g_, leading to a temporally optical state. Once the temperature nears the *T*_g_ again, the polymer will recover to the original shape and structural color. The time factor is a consequence of the slow kinetics that are associated with the temperature‐induced morphological transformation of the polymer, ultimately resulting in a slow irreversible optical change when exposed to temperatures at or slightly above *T*_g_.^[^ ^34^ ^]^

**Figure 4:**
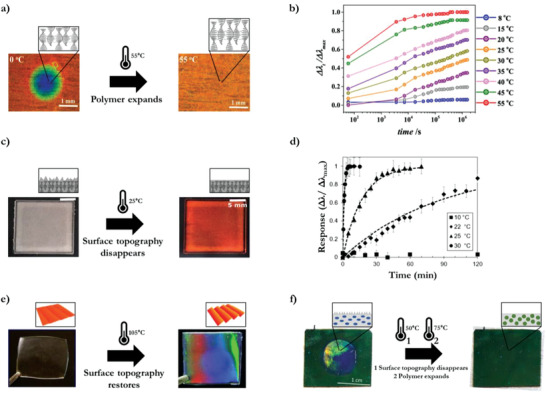
Shape memory structural colored polymers: optical temperature indicators and TTIs. a) Thermal shape memory recovery over time showing a red shift during polymer expansion of the deformed cholesteric LC shape memory polymer. b) Time‐dependent optical response to isothermal temperature exposure (8–55 °C). Adapted with permission.^[^ ^36^ ^]^ Copyright 2017, American Chemical Society. c) Thermal shape memory recovery over time of an embossed scattering topography surface to a flat surface at isothermal exposure to 25 °C. d) Time‐dependent optical response to isothermal temperature exposure (10, 22, 25, and 30 °C). Adapted with permission.^[^ ^38^ ^]^ Copyright 2019, Wiley‐VCH. e) Thermal shape memory recovery of a diffractive surface topography. Adapted with permission.^[^ ^7^ ^]^ Copyright 2013, Wiley‐VCH. f) Dual thermal shape memory recovery, the first step is the recovery of the embossed binder (diffractive topography disappears), the second step is the recovery of the compressed cholesteric LC particles. Adapted with permission.^[^ ^39^ ^]^ Copyright 2020, Wiley‐VCH.

Cholesteric LC shape memory polymer‐based TTIs have been demonstrated by compressing the polymer throughout the thickness, this decreases the photonic layer spacing and therefore the reflected color is blue shifted from red to blue.^[^ ^35^ ^]^ The onset temperature and the sensitivity of the thermal shape memory recovery can be tuned by altering the polymer network properties through incorporation of another polymer resulting in a semi‐interpenetrating network which results in a broader *T*_g_ (**Figure** **4**a). The kinetic study of the original structural color recovery showed that the rate of change decreased continuously until an equilibrium state is reached after ≈1 h at isothermal temperature after heating from 0 °C to a specific temperature *T* (8 °C ≤ *T* ≤ 55 °C). The irreversible color change is proportional to the difference between the upper limit of the *T*_g_ and the temperature of exposure (Figure 4b).^[^ ^36^ ^]^

Blue phase LC shape memory polymers can also be programmed with a pressure‐dependent blue shift to fabricate optical integrators. The full recovery of the original color through a red shift takes 1 h at 40 °C and only 10 s at 80 °C.^[^ ^37^ ^]^

Instead of compressing a polymer throughout the entire volume, a surface topography can be embossed that causes scattering of the light reflected by the LC polymer due to the plasticity of the photonic shape memory polymer above *T*_g_. This scattering induces a white reflection that transitions back to the reflected color when the flat surface restores due to exposure above *T*_g_ (Figure 4c). At 25 °C, full recovery takes 1 h while at 30 °C it takes only 4 min (Figure 4d).^[^ ^38^ ^]^

Another approach does the opposite by flattening the surface of a shape memory polymer with a scattering or diffractive microstructure topography. This method programs a temperature‐dependent transition from a transparent to a diffractive polymer (Figure 4e).^[^ ^7^ ^]^ The research shows that for this transition there is no time dependence when the diffractive surface restores during a temperature exposure at 105 °C.

The latter two methods have been combined in a polymer coating that contains cholesteric LC particles in a polymer binder.^[^ ^39^ ^]^ As both the polymer binder and the cholesteric LC particles have a different and specific *T*_g_, two distinct irreversible temperature responses (at 50 and 75 °C) are obtained by compressing the particles and by embossing the binder with a diffractive surface topography (Figure 4f). This system was not characterized for a specific time response, but it promises tunability as both the particles and the binder *T*_g_ can be modified for the time and temperature response of an optical integrator.

**Figure 5:**
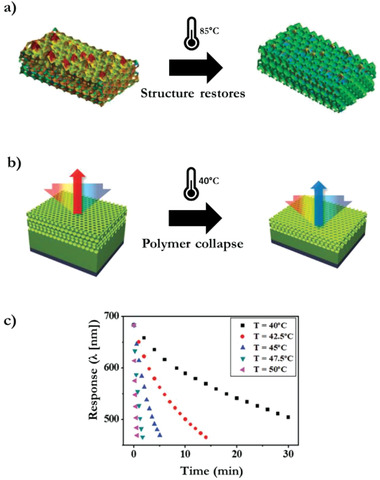
Inverse opal structural color TTI. a) Thermal shape memory recovery of the color reflecting polymer structure after exposure to 85 °C. Adapted with permission.^[^ ^42^ ^]^ Copyright 2015, Wiley‐VCH. b) Temperature‐dependent polymer collapse of an inverse opal structure, resulting in a blue shift of the optical response. c) Time‐dependent optical response to isothermal temperature exposure (40–50 °C). Adapted with permission.^[^ ^41^ ^]^ Copyright 2019, Wiley‐VCH.

An inverse opal structure embedded in a shape memory polymer system facilitates a temperature‐induced recovery of the photonic structure, equivalent as applied in LC polymers (**Figure** **5**a). Contrarily, this system has a short response time (<2 min), caused by the sharp glass transition temperature.^[^ ^40^ ^]^

Inverse opals imprinted in a different polymer system can record temperature exposure through irreversible creep deformation and report the history by a blue shift due to the collapse of the photonic film, changing the reflected color from red to blue (Figure 5b). The optical response is time‐dependent to temperatures above 40 °C as the mechanical strength of the polymer incrementally decreases (Figure 5c).^[^ ^41^ ^]^ Additionally, the crosslinking density can be regulated through the UV intensity applied during polymerization, which allows to produce an array that decouples the temperature and time of an isothermal exposure.

**Figure 6:**
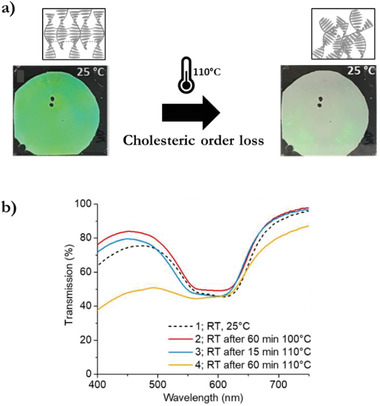
TTI based on cholesteric order loss of a supramolecular crosslinked LC polymer a) coating before and after exposure to 110 °C (<*T*_iso_) for 1 h. b) Time‐ and temperature‐dependent optical response to isothermal temperature exposure represented by the spectroscopic transmission band change. Adapted with permission.^[^ ^43^ ^]^ Copyright 2020, American Chemical Society.

LC polymers are characterized by a nematic to isotropic transition temperature (*T*_NI_) which was exploited in a TTI cholesteric polymer having only supramolecular crosslinks. Exposing the photonic polymer to temperatures above the isotropic transition yields a disordered state over time as the linear polymers transgress their cholesteric orientation. The resulting coating is scattering at room temperature as it reflects the full‐wave spectrum because of the lack of orientational order in the cholesteric phase (**Figure** **6**a).^[^ ^43^ ^]^ This transition is time dependent due to the dynamic supramolecular hydrogen bonds that slow down the order loss. After 60 min at 110 °C, the coating obtains a scattering white color (Figure 6b).

## 3 Chemical‐Responsive Optical Indicators

### 3.1 Chemicals in Solution

**Figure 7:**
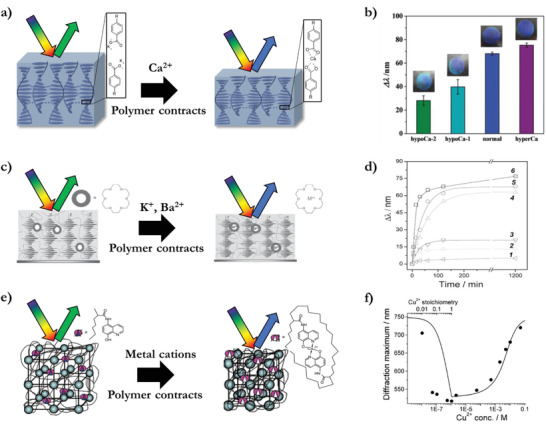
Cation indicators. a) Ca^2+^ cation carboxylate complexation from solution in a potassium salt cholesteric LC polymer causes a contraction, which results in a blue shift. b) Concentration‐dependent optical response for Ca^2+^ in normal serum, hypocalcemia (hypoCa‐1 and hypoCa‐2), and hypercalcemia (hyperCa) samples. Adapted with permission.^[^ ^44^ ^]^ Copyright 2016, Wiley‐VCH. c) Potassium and barium cations complexation by a crown ether incorporated in a cholesteric LC polymer and causes a contraction, which results in a blue shift. d) Kinetic‐ and concentration‐dependent optical response for different barium perchlorate water solutions with concentration: 1) 0 m, 2) 0.0001 m, 3) 0.001 m, 4) 0.005 m, 5) 0.01 m, 6) 0.02 m. Adapted with permission.^[^ ^45^ ^]^ Copyright 2012, Wiley‐VCH. e) Cu^2+^ and other bivalent metallic cations complexation by 8‐hydroxyquinoline: At low metal concentrations the cations form bis‐liganded complexes which crosslinks and causes the hydrogel to contract, which results in a blue shift. f) Concentration‐dependent optical response for Cu^2+^ saline buffer solution, demonstrating a blue shift at low concentrations, and a red shift at higher concentrations. Adapted with permission.^[^ ^46^ ^]^ Copyright 2003, American Chemical Society.

Ion detection in solution has been demonstrated with structural colored polymers by complexation to recognize and retain specific cation concentrations. One method converts a cholesteric LC polymer with carboxylic acid side chain end groups into hygroscopic potassium carboxylate polymer after base treatment with KOH. This monovalent salt causes water absorption. Exposure of the salt polymer to bivalent cations in solution, such as Ca^2+^, leads to ion exchange and the formation of calcium carboxylate crosslinks. This crosslinking contracts the polymer and therefore results in a calcium concentration‐dependent blue shift (**Figure** **7**a).^[^ ^44^ ^]^ Such an optical response functions as an indicator for calcium levels in blood serum as the sensitivity is in the millimolar concentration, comparable to the total concentration of calcium in blood (Figure 7b). The indicator is recovered by using a strong acid to remove the bivalent salt complex, followed by a base treatment with KOH to form a monovalent salt again.

Another approach to obtain ion retention is by the implementation of crown ethers which form a complex with specific cations. Polymer‐stabilized cholesteric LC composites containing 18‐crown‐6 ether captures cations from a salt solution, again resulting in a blue shift of the photonic polymer. For this system, complexation with potassium and barium ions was obtained and compared (Figure 7c).^[^ ^45^ ^]^ A time‐dependent effect is observed during exposure, due to the polymer density and the slow complex formation (Figure 7d).

Besides crown ethers, 8‐hydroxyquinoline embedded as polymer side groups in an IOH detects metallic cations Cu^2+^, Zn^2+^, and Co^2+^ in aqueous media through complexation (Figure 7e).^[^ ^46^ ^]^ At ultralow concentrations (<µM), the cations form bisliganded crosslinks that contract the polymer, which results in an irreversible blue shift as the complex is energetically favored. For these low concentrations, the time necessary to reach equilibrium took up to 6 h. At higher cation concentrations, the bisliganded complexes are in a concentration‐dependent equilibrium with the formation of monoliganded cation complexes, causing a red shift through the breaking of the bisliganded crosslinks (Figure 7f). Based on the interaction of cations with similar 8‐hydroxyquinoline association constants, this type of optical indicator can be used to detect metals in drinking water.

**Figure 8:**
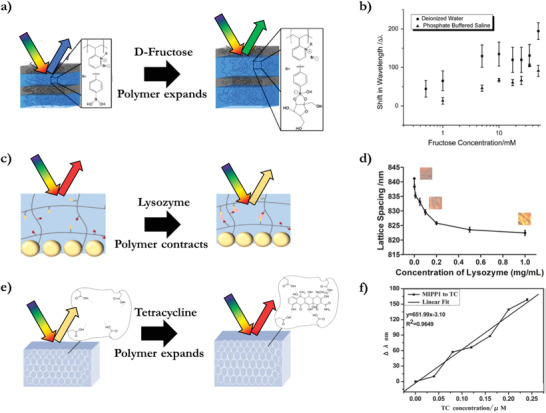
Biomolecule indicators. a) Fructose binding with boronic acid causing the BCP polymer to expand and redshift. b) Concentration‐dependent optical response for fructose solution in water and in PBS buffer. Adapted with permission.^[^ ^48^ ^]^ Copyright 2013, Elsevier. c) Lysozyme interaction with carboxylic acid groups and hydrophobic groups in a responsive hydrogel causing the hydrogel and the 2D colloidal array to contract, resulting in a blue shift. d) Concentration‐dependent optical response for lysozyme in urine solution. Adapted with permission.^[^ ^49^ ^]^ Copyright 2020, Elsevier. e) Tetracycline complexation in a molecularly templated inverse opal hydrogel causing the polymer to expand and redshift. f) Concentration‐dependent optical response for tetracycline in aqueous solution. Adapted with permission.^[^ ^50^ ^]^ Copyright 2012, Royal Society of Chemistry.

Detection of biomolecules allows for more versatile approaches as molecules have more distinct features such as molecular volume and different functional groups in contrast to cations. Phenylboronic acid incorporated in an acrylamide IOH can form covalent complexes with glucose. This complexation increases the volume of the hydrogel, resulting in a red shift.^[^ ^47^ ^]^ Similarly, fructose is selectively complexated by phenylboronic acid in a self‐assembled lamellar BCP^[^ ^48^ ^]^ (**Figure** **8**a). The binding of fructose, a 1,2 cis diol, is preferred over binding with glucose, mannose, or galactose which are 1,3 cis diols. The concentration‐dependent optical response demonstrated a sensitivity of 0.5 × 10^−3^ m of fructose in phosphate‐buffered saline (PBS) buffer solution (Figure 8b). A 2D colloidal polymer monolayer on top of a hydrogel allows for sensitive and fast indicators for biomolecules as the hydrogels are prone to a volume change, resulting in a change in the lattice spacing of the colloidal monolayer. Through an optimal chemical composition of a stimuli‐responsive hydrogel, a range of biomolecules can be detected. One example features acrylic acid as a hydrophilic monomer and *N*‐*tert*‐butylacrylamide as a hydrophobic monomer in an acrylamide hydrogel that electrostatically binds lysozyme through both hydrophobic and hydrogen bonding interactions, resulting in a shrinkage of the hydrogel and a lattice spacing decrease of the 2D colloidal polymer monolayer (Figure 8c).^[^ ^49^ ^]^ The concentration‐dependent optical response has a limit of detection (LOD) of 1.4 mg L^−1^, and the detection worked in practical solutions like artificial tears and urine (Figure 8d). The indicator is reusable after a thermal treatment that results in the release of the lysozyme.

Another approach to obtain a molecular specific retention is by molecular imprinting of a template molecule in the hydrogel through complex formation. After a removal procedure of the template molecule, the obtained polymer possesses specific nanocavities complementary to the target compound. This provokes a selective response based on both volumetric and chemical interactions that results in retention of a molecule in the cavity.

Molecular imprinting of (oxy)tetracycline as a hydrogen bonded template in acrylamide and acrylic acid, forms an IOH indicator. After removal of the template, the polymer holds specific binding sites for tetracycline‐based antibiotics (Figure 8e).^[^ ^50^ ^]^ Due to the absorption of the target molecules, the hydrogel expands which causes a concentration‐dependent red shift response, with a detection limit in the µM range (Figure 8f). This mechanism is implemented in a hydrogel indicator with a 2D colloidal polymer monolayer on top of a hydrogel which allows for fast screening of antibiotics in milk and honey.^[^ ^51^ ^]^ A similar example is the detection of sulfonamide antibiotics in egg whites through molecular templating of an IOH again through complexation in an acrylamide–acrylic acid polymer.^[^ ^52^ ^]^ Here, the indicator demonstrates a blue shift response, due to a high degree of crosslinking in the recognition cavities which causes shrinking of the IOH volume. This indicator can be reused after washing with acetic acid/methanol/sodium dodecyl sulfate mixture and deionized water.

The attainable selectivity and sensitivity in molecular response is illustrated by the red shift of a methacrylic acid IOH indicator. The polymer expansion is caused by chiral recognition of 0.01 × 10^−3^ m of the amino acid L‐pyroglutamic acid in a monosodium glutamate solution.^[^ ^53^ ^]^

The biomolecules, hemoglobin, bovine serum albumin, and horseradish peroxidase were separately imprinted to form an array of acrylamide IOH beads with an angular independent color. The macropores of the beads are interconnected and provide greater surface area and more interaction sites, but also offer easier access for the analytes to the interaction sites to introduce fast absorption and swell resulting in a red shift. A multiplex array system can be used for detecting multiple biomolecules at once in medical diagnostics.^[^ ^54^ ^]^

### 3.2 Solvents

Solvent indicators based on polymer swell are sensitive to evaporation,^[^ ^55^ ^]^ and only retain the response after exposure for solvents with a high boiling point.^[^ ^56^ ^]^ Therefore, other mechanisms are employed to ensure that indicators remain in an invariable structural colored state after solvent exposure.

**Figure 9:**
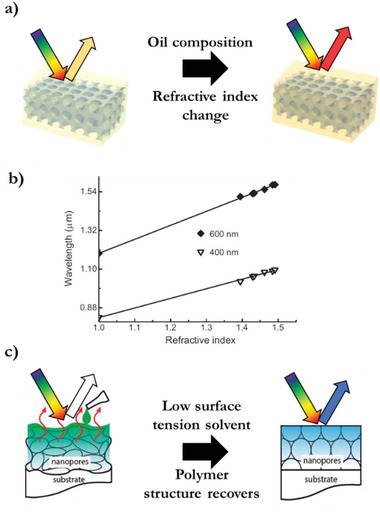
Solvent indicators. a) Oil composition determines the refractive index difference between the pores and the inverse opal polymer which causes a redshift when the refractive index difference increases. b) Refractive index‐dependent optical response of different adsorbed oil mixtures. Adapted with permission.^[^ ^57^ ^]^ Copyright 2008, Wiley‐VCH. c) Low surface tension solvent‐induced shape memory recovers the photonic structure. Adapted with permission.^[^ ^58^ ^]^ Copyright 2018, American Chemical Society.

An inverse opal polymer indicator based on refractive index changes is fabricated based on a phenolic resin with both superoleophilic and superhydrophobic properties (**Figure** **9**a). The reflected wavelength shift upon sorption of oils, is a linear function of the refractive index of the adsorbed oil (Figure 9b). This forms an indicator for oil and petroleum derivatives and mixtures thereof. Recycling is done by desorption of the oil in octane, after which the octane is evaporated.^[^ ^57^ ^]^

Inverse opal shape memory polymers can have an irreversible optical response triggered by a difference in solvent polymer interaction which restores a collapsed polymer structure. The polymer structure transformation is dependent on the elastic modulus of the polymer and the solvent surface tension (Figure 9c). This enables solvent evaporation‐induced shape memory deformation and solvent swelling triggered shape memory recovery, all occurring at room temperature.^[^ ^58^ ^]^ By varying the compositions of their constituent polymers, the elastic moduli of the polymer can be modulated, leading to different reflected colors in response to different solvents, such as water, ethanol, and acetonitrile. A similar approach demonstrates the ability to detect trace amounts of a swelling solvent in a nonswelling solvent by monitoring the apparent color changes associated with the swelling‐induced shape memory recovery.^[^ ^59^ ^]^ This high sensitivity also allows for vapor‐based detection by the rapid diffusion of solvent molecules in the deformed polymer chains which triggers instantaneous shape memory recovery of the macropores with nanoscopic wall thickness.

### 3.3 Vapors

**Figure 10:**
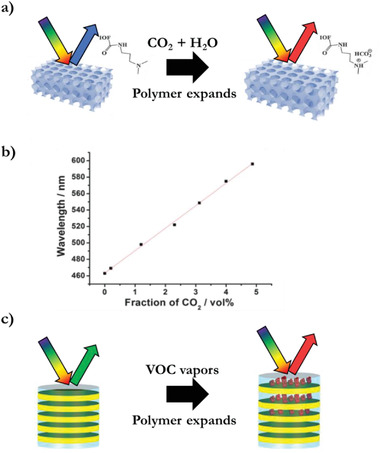
Vapor indicators and integrators. a) CO_2_ gas reacts with amine and water to form an ion pair in an inverse opal hydrogel polymer which induces expansion and results in a red shift. b) Concentration‐dependent optical response for CO_2_ in CO_2_–N_2_ mixtures. Adapted with permission.^[^ ^60^ ^]^ Copyright 2013, The Royal Society of Chemistry. c) Volatile organic compound vapors induce the formation of cocrystals within the photonic polymer stack. The polymer expands, resulting in a red shift. Adapted with permission.^[^ ^61^ ^]^ Copyright 2016, American Chemical Society.

An amine‐functionalized inverse opal polymer indicator is able to track the presence of CO_2_ gas over the whole concentration range (0–100%) by a full‐color response with a small amount of gas sample.^[^ ^60^ ^]^ After the reaction of CO_2_ and H_2_O with the amine, an ion pair is formed which induces water absorption (**Figure** **10**a). At low concentrations, a concentration‐dependent red shift optical response is observed from 0 to 4.9 vol% (Figure 10b). The detection limit for the gas mixture was 0.2 vol%. This approach provides a sensitive, quantitative, and interference tolerant method for tracking CO_2_. The indicator can be reset by washing with a basic solution. Other acidic and basic gases could also be detected by changing the functional groups in the photonic hydrogel.

A photonic polymer stack of poly(*p*‐phenylene oxide) and cellulose acetate demonstrates vapor selectivity attained by specific interactions between the polymer and analytes. The interactions drive the analyte intercalation through the uptake of volatile organic compound (VOC) vapors which induces the formation of semicrystalline nanoporous phases, resulting in distinct optical properties.^[^ ^61^ ^]^ After desorption of the VOC vapors, the crystalline phase remains unchanged. The analysis of the response kinetics also allows to distinguish binary mixtures of benzene, 1,2‐dichlorobenzene, carbon tetrachloride, and toluene (Figure 10c). The slow vapor diffusion throughout the polymer layers combined with the crystallization process results in a time dependence up to 40 min, classifying this material as an integrator.

Additionally, such optical integrators can be used to determine the diffusion coefficients of small molecules within polymer materials. A photonic stack comprising polystyrene (PS) and cellulose acetate (CA), is able to discriminate between air enriched with different short‐chain alcohols, including methanol, ethanol, 1‐propanol, 2‐propanol, and 1‐butanol, and their binary mixtures.^[^ ^62^ ^]^ This introduces a low‐cost optical detection method integrable to in situ assessment of barrier polymers used for the encapsulation of optoelectronic devices, food packaging, and goods storage in general.

A different polymer stack comprising Hyflon, a commercial fluorinated polymer, and a nonfluorinated polymer with a different refractive index, allows for vapors of perfluorinated species to become detectable as the Hyflon polymer layers act as the active and selective medium for sensing. Again, these integrators show a concentration and time (up to 100 min) dependent behavior.^[^ ^63^ ^]^

## 4 Pressure‐Responsive Optical Indicators

**Figure 11:**
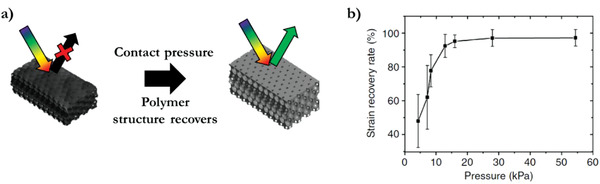
Pressure indicator and light integrator. a) Contact pressure promotes recovery of the photonic inverse opal structure due to pull‐off force caused by van der Waals interactions. This restores the reflection properties; the material goes from transparent to a green reflection. b) Pressure‐dependent optical response, 30 kPa is required to completely recover the photonic structure. Adapted with permission.^[^ ^64^ ^]^ Copyright 2015, Macmillan Publishers Limited.

An inverse opal shape memory polymer demonstrates a pressure response that restores the photonic polymer structure. The polymer shape memory system is cold programmed through the structural deformation caused by the capillary pressure generated during water evaporation. The recovery of the photonic inverse opal structure is due to the pull off force after a contact pressure (**Figure** **11**a). The degree of recovery shows a pressure dependence, up to 30 kPa, and is stable after the contact moment (Figure 11b).^[^ ^64^ ^]^

## 5 Light‐Responsive Optical Indicators

The incorporation of a light‐responsive azobenzene dye into cholesteric polymer particles allows for a photothermal recovery of the particles after compressing in a shape memory programming step. The different intensities of UV light result in temperature control and as such allows for a proportional recovery. The equilibrium shift during illumination allows for an intensity‐dependent shift over time, resulting in optical integrators. Additionally, the light beam allows for spatial control, allowing for local recovery of the original reflection.^[^ ^39^ ^]^

**Figure 12:**
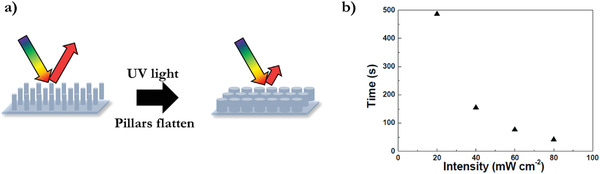
UV light induces a change in the geometry of the polymer pillars due to isomerization of azobenzene in the LC polymer network. The 2D pillar array flattens and therefore the reflection intensity decreases. b) Intensity‐dependent response time to reach the maximum response upon UV light exposure. Adapted with permission.^[^ ^65^ ^]^ Copyright 2012, Wiley‐VCH.

A light‐responsive 2D microarray of cholesteric LC polymer was made by replica molding. The photoisomerization of the azobenzene groups resulted in deformation of the polymer pillars.^[^ ^65^ ^]^ As such, the polymer microarray showed a decrease in the intensity of the reflection spectra after UV light irradiation caused a change in the order of the microarray (**Figure** **12**a). The deformation time of the pillars shows to be intensity dependent (Figure 12b) and can be recovered by visible light (530 nm).

Furthermore, a comparable cholesteric LC polymer with an embedded azobenzene was applied in an inverse opal structure to fabricate a dual‐responsive photonic polymer. The inverse opal polymer showed a deformation of the structure in response to UV light, which causes a decrease in reflection intensity. This change in the periodic structure is ascribed to the contraction of the polymer induced by the photochemical reactions of the azobenzene moieties or the thermal‐induced phase transition.^[^ ^66^ ^]^ Again by visible light, the original polymer structure can be restored. Additionally, heating this system can also cause a temperature‐dependent structure deformation caused by collapse of the porous polymer structure above *T*_g_.

## 6 Conclusion and Prospect

The optical response of indicators based on structural colored polymers of all types is described in this review. **Table** **1** provides an overview of the discussed indicators and integrators, categorized on the polymer type with a characterization of the responsivity (TTI, chemical indicator, pressure indicator, light indicator), an indication of the sensitivity, and the response mechanism that is applied.

**Table 1:** Optical indicators and integrators based on structural colored polymers

Polymer type	Responsivity	Sensitivity	Response mechanism	Ref.
Cholesteric LC polymer	TTI	>0 °C	Evaporation of water	^[^ ^25^ ^]^
Cholesteric LC polymer	TTI	8–55 °C	Shape memory recovery	^[^ ^35^, ^36^ ^]^
Blue phase LC polymer	TTI	8–55 °C	Shape memory recovery	^[^ ^37^ ^]^
Cholesteric LC polymer	TTI	20–30 °C	Shape memory recovery of topography	^[^ ^38^ ^]^
1D diffraction topography	TTI	105 °C	Shape memory recovery of topography	^[^ ^7^, ^34^ ^]^
Cholesteric LC polymer particles in binder	Dual TTI	50 °C + 75 °C	Shape memory recovery	^[^ ^39^ ^]^
Inverse opal polymer	TTI	40 °C	Temperature‐induced structure collapse	^[^ ^41^ ^]^
Cholesteric LC polymer	TTI	105 °C	Order loss above *T*_iso_	^[^ ^43^ ^]^
Cholesteric LC polymer	Ca^2+^ indicator	0.1 m	Ion exchange contracts polymer	^[^ ^44^ ^]^
Cholesteric LC polymer	K^+^ and Ba^2+^ indicator	0.1 × 10^−3^ m	Cation complexation induces polymer contraction	^[^ ^45^ ^]^
Inverse opal hydrogel	Pb^2+^ indicator	<10^−6^ m	Cation complexation induces polymer contraction	^[^ ^46^ ^]^
Inverse opal hydrogel	Glucose indicator	5 × 10^−3^ m	Glucose complexation induces polymer expansion	^[^ ^47^ ^]^
Lamellar block copolymer	Fructose indicator	0.5 × 10^−3^ m	Fructose complexation induces polymer expansion	^[^ ^48^ ^]^
2D colloid monolayer on hydrogel	Lysozyme indicator	1.4 mg L^−1^	Complexation induces polymer expansion	^[^ ^49^ ^]^
Inverse opal hydrogel	Tetracycline indicator	0.04 × 10^−6^ m	Absorption in molecular template induces polymer expansion	^[^ ^50^ ^]^
2D colloid monolayer on hydrogel	Tetracycline indicator	10 × 10^−6^ m	Absorption in molecular template induces polymer expansion	^[^ ^51^ ^]^
Inverse opal hydrogel	Sulfonamide indicator	3.8 × 10^−6^ m	Absorption in molecular template induces polymer contraction	^[^ ^52^ ^]^
Inverse opal hydrogel	Amino acid indicator	10 × 10^−6^ m	Absorption in molecular template induces polymer expansion, with chiral recognition	^[^ ^53^ ^]^
Inverse opal hydrogel	Biomolecule indicator	1 µg L^−1^	Absorption in molecular template induces polymer expansion	^[^ ^54^ ^]^
Inverse opal polymer	Oil composition indicator	0.02 RI change	Refractive index difference influences reflection	^[^ ^57^ ^]^
Inverse opal polymer	Solvent indicator	–	Shape memory recovery triggered by specific solvent swell (water, ethanol, acetonitrile)	^[^ ^58^ ^]^
Inverse opal polymer	Solvent indicator	150 ppm	Shape memory recovery triggered by trace amount solvent (ethanol)	^[^ ^59^ ^]^
Inverse opal hydrogel	CO_2_ indicator	1 vol%	Chemical reaction forms an ion pair, induces polymer expansion	^[^ ^60^ ^]^
Multilayer polymer stack	VOC integrator	Saturated air	VOC‐induced crystallization, induces polymer expansion	^[^ ^61^ ^]^
Multilayer polymer stack	Alcohol vapors integrator	0.8 mg L^−1^	Diffusion and absorption based on analyte‐polymer affinity, induces polymer expansion	^[^ ^62^ ^]^
Multilayer polymer stack	Perfluorinated vapors integrator	–	Diffusion and absorption based on analyte‐polymer affinity, induces polymer expansion	^[^ ^63^ ^]^
Inverse opal polymer	Pressure indicator	–	Pull of force after contact pressure recovers polymer structure	^[^ ^64^ ^]^
Cholesteric LC polymer particles	UV light integrator	150 mW cm^−2^	Shape memory recovery triggered by photothermal effect	^[^ ^39^ ^]^
2D pillar array of LC polymer	UV light integrator	20 mW cm^− 2^	Azobenzene photoisomerization induced deformation, reduces reflection intensity	^[^ ^65^ ^]^
Inverse opal LC polymer	UV light integrator	50 mW cm^− 2^	Azobenzene photoisomerization induced deformation, reduces reflection intensity	^[^ ^66^ ^]^

A prominent class of responsive photonic polymer integrators is TTIs, which allow to program a time‐dependent change during temperature exposure. The origin of the time dependence lies in the mobility of polymer chains determined by the *T*_g_ and polymer interactions. This allows for a range of polymer modifications to tune the time and temperature response in these integrators. A broad extend of temperature sensitivity and time frames has been reported, however a polymer‐based TTI with the exact specifications (between 5 and 10 °C) to monitor thermal food degradation over time for cold chain management has not been demonstrated with structural colored polymers yet.^[^ ^67^ ^]^ Printable TTI labels could provide an autonomous optical indicator with a facile readout to track perishable or temperature‐sensitive consumables, such as food and pharmaceuticals, during transport or their respective lifetime.^[^ ^68^ ^]^ As a more accurate alternative for static expiration dates, these indicators have the potential to decrease waste of consumables. The demonstrated TTIs based on shape memory allow programming or reconfiguration of the responsive systems which implies they are reusable. One temperature‐dependent indicator that demonstrates application required specifications, is an LC indicator to verify steam sterilization conditions.^[^ ^43^ ^]^ This validates the potential of these materials to match application requirements.

Most chemical‐responsive systems retain chemicals by ionic interactions or complexation, which also gives the advantage of reconfiguration that is often highlighted. The optical response is dependent on the concentration of a chemical in solution, solvent, or gas. The sensitivity of these systems depends on the number of reaction sites for the analyte, which can be determined through the composition. By embedding specific chemical or supramolecular interactions, cross‐sensitivity can be excluded. However, all responsive polymer systems are intrinsically temperature dependent. Other than diffusion and complexation kinetics, there is not much reported on (the control over) the time‐dependent response. These indicators, which are able to detect chemicals in solutions, can facilitate fast diagnostics of health parameters by measuring salt concentrations, metal cations, amino acids, and other biomolecules. Therefore, they have the potential to serve as personal care aids in providing an accurate tool to follow‐up, e.g., glucose concentrations in diabetes patients.

Vapor indicators apply molecular affinity to retain the gas inside the polymer system, another approach is vapor‐induced crystallization which both results in polymer expansion. Reliable detection of certain gases is vital for human safety in hazard situations, for which optical indicators can track and record conditions continuously. For pressure or strain‐responsive polymer indicators, only one optical polymer system was found in literature despite the growing demand for impact detection during transport of goods.^[^ ^69^ ^]^ Such indicators allow to trace back an impact on a surface, which is interesting for security features or for tracking safe transport conditions. Potentially, nonelastic polymers are able to maintain the deformation in a photonic structure in the development of novel strain indicators.

Light‐responsive behavior can be implemented in polymers by certain molecules that have a mechanical or thermal effect when illuminated. Common molecular switches are azobenzene, spiropyran, or bi‐2‐naphtol derivatives for their photoisomerization properties. An interesting application is demonstrated by a nonpolymerized structural color UV indicator able to monitor the exposure to harmful sunlight.^[^ ^70^ ^]^ Implementation in an optical integrator can result in a mechanical deformation as a response to light. For light‐responsive integrators, further control of the time factor is possible as the response for a specific photoswitch molecule is dependent on an isomerization equilibrium.

Polymer indicators with an irreversible response to other stimuli such as electricity, humidity, magnetism, and tensile force have not been reported. There are also opportunities to design novel indicator response types such as indicators that display an irreversible optical response based on a permanent change in the refractive index difference within the structure. Furthermore, an absorption gradient can be created that broadens the response time. Some examples already demonstrated a color loss or a scattering effect instead of a color shift, this can serve as an inspiration to develop novel response types based on an irreversible degradation or loss of order. Another promising new type of indicators is based on blue phase LC polymers. The intricate photonic structure obtained in blue phase LC polymers has a lot of potential for indicators as a small structural change will result in a big optical response, therefore a high sensitivity can be reached.

The manufacturing of structural colored polymers is based on self‐assembly as a bottom‐up approach, which allows for scalability of production. However, some indicator types require additional processing such as etching or an activation treatment. Therefore, printable materials that can be considered as “indicator inks” are most desired for future applications. The rise of new top‐down manufacturing methods such as 4D additive manufacturing, promises a further increase in tunability and response modes for indicators and integrators as it enables innovative structural transitions as well as an increased response through printing porous polymer structures.^[^ ^71^ ^]^

**Figure 13:**
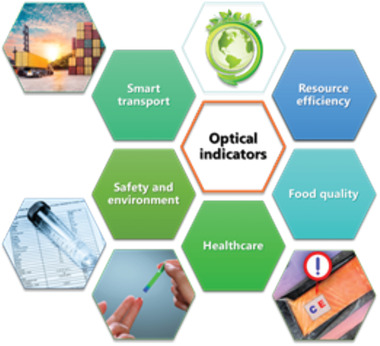
Potential impact of optical indicators. Adapted with permission.^[^ ^44^, ^73^ ^]^ Copyright 2012, Wiley‐VCH; Copyright 2020, Jose‐Luis Olivares, MIT.

A change in visible color serves as a practical way of interpretation for both the human eye and optical electronic readout, with the possibility to derive the exposure amount equivalent to the color difference. The potential applications promise to have a great impact on the societal challenges as formulated in the Horizon Europe Research and Innovation program (**Figure** **13**).^[^ ^72^ ^]^ Easy to interpret visual indicators can contribute to a more conscious storage and consumption management for pharmaceuticals and food products, which consequently reduces the production and the burden on valuable resources. Furthermore, simplified monitoring of health conditions at home is possible by novel fluid indicators which increases observation while lowering the workload of medical services. Also, for personal safety and monitoring environmental conditions such as gas exposure, polluted water, or precarious (sun)light exposure, visual indicators might offer a convenient detection tool.

To summarize, the versatility allowed by the different structural colored optical indicators has enabled access to monitor a wide range of physical and chemical exposure conditions. These systems allow tunability on a molecular level to modify the response (range) and implement reconfiguration. Through molecular engineering of the photonic nanostructures, interactions based on supramolecular principles within the polymer system allow for high selectivity and sensitivity. Until now, many concepts have been established which have potential for a specific application in healthcare, food quality, safety, or transport. Future research will tailor existing systems to match the exact needs for specific applications, possibly by new manufacturing methods and principles. Next to that, there is a lot of potential for innovative systems that combine new molecular triggers, novel response mechanisms, or cross over principles from other (structural colored) polymer systems.

## Conflict of Interest

The authors declare no conflict of interest.
